# Genetic Characterization of Extensively Drug-Resistant *Shigella sonnei* Infections, Spain, 2021–2022

**DOI:** 10.3201/eid2911.221746

**Published:** 2023-11

**Authors:** Camille Jacqueline, Guillermo Ruiz Carrascoso, José Gutiérrez-Fernández, Teresa Vicente Rangel, Lidia Goterris, Fernando Vazquez Valdes, Domingo Fernández Vecilla, Matilde Elía López, Maria Rocío Martinez Ruiz, Carmen Aspiroz Sancho, Ramón Perez Tanoira, Elia Sirvent Quílez, Alba de la Rica Martínez, Nieves Gonzalo Jiménez, Cristina García Salguero, Eva González Barbera, Maria Reyes Sánchez Florez, Francisco J. Merino, Begoña Sagardia Redondo, Enrique Rodriguez Guerrero, Claudia Sanz González, Silvia Herrera-Leon

**Affiliations:** European Public Health Microbiology Training Program, European Centre for Disease Prevention and Control, Stockholm, Sweden (C. Jacqueline);; Centro Nacional de Microbiología, Instituto de Salud Carlos III, Majadahonda, Spain (C. Jacqueline, S. Herrera-Leon);; Servicio de Microbiología Clínica, Hospital Universitario La Paz, Madrid, Spain (G.R. Carrascoso, C.S. González);; Universidad de Granada, Granada, Spain (J. Gutiérrez-Fernández);; Hospital General Universitario Gregorio Marañon, Madrid, Spain (T.V. Rangel);; Hospital Universitario Vall d'Hebron, Barcelona, Spain (L. Goterris);; Hospital Universitario Central De Asturias, Oviedo, Spain (F.V. Valdes);; Hospital Universitario de Basurto, Bilbao, Spain (D.F. Vecilla);; Hospital Universitario de Navarra, Pampona, Spain (M.E. López);; Hospital Puerta De Hierro, Majadahonda, Spain (M.R.M. Ruiz);; Hospital Royo Villanova, Zaragoza, Spain (C. Aspiroz Sancho);; Hospital Universitario Príncipe de Asturias, Alcalá de Henares, Spain (R.P. Tanoira);; Hospital General Universitario de Elche, Alicante, Spain (E.S. Quílez, A.R. Martínez, N.G. Jiménez);; Hospital Clínico San Carlos, Madrid, Spain (C.G. Salguero);; Hospital Universitario y Politécnico La Fe, Valencia, Spain (E.G. Barbera);; C.H.U. Nuestra Señora de Candelaria, Santa Cruz de Tenerife, Spain (M.R.S. Florez);; Hospital Universitario Severo Ochoa, Leganés, Spain (F.J. Merino);; Laboratorio De Salud Pública, Palma De Mallorca, Spain (B.S. Redondo);; Hospital Universitario Virgen de las Nieves, Granada, Spain (E.R. Guerrero)

**Keywords:** *Shigella sonnei*, drug resistance, Spain, sexual transmission, infections, bacteria, antimicrobial resistance, XDR, extensively drug-resistant

## Abstract

In 2022, the United Kingdom reported an increase in drug resistance in *Shigella sonnei* isolates. We report 33 cases in Spain genetically related to the UK cases and 4 cases with similar antimicrobial resistance profiles infected with genetically distant strains. Our results suggest circulation of multiple genetic clusters of multidrug-resistant *S. sonnei* in Spain.

On January 27, 2022, the United Kingdom reported an increased number of infections with extensively drug-resistant (XDR) *Shigella sonnei* during September 1, 2021–January 10, 2022 (*1*). A total of 146 cases were later reported in 9 other countries in Europe, all having either a similar XDR profile or close genetic relationship to the UK cases (*2*). Most cases were linked to sexual transmission between gay, bisexual, and other men who have sex with men (MSM) (*2*). 

In Spain, information on *S. sonnei* exposure is rarely available (only in 7.2% of cases), but person-to-person transmission is the most frequently observed. Previous studies in Spain reported circulation of lineages of *S. sonnei* resistant to first- and second-line oral treatments among MSM in different autonomous communities (Catalunya, Andalucía, País Vasco, and Madrid) as early as 2015 (*3*–*7*). We describe the multidrug-resistant (MDR) isolates of *S. sonnei* circulating in Spain during January 2021–April 2022.

## The Study

For this investigation, we defined a suspected case as a patient with laboratory-confirmed *S. sonnei* infection; an MDR profile characterized by nonsusceptibility to >1 agent in >3 of the antimicrobial categories tested, including third-generation cephalosporins, aminoglycosides, sulfamethoxazole, and fluroquinolones; and a specimen collected in Spain during January 1, 2021–April 1, 2022. Over the study period, hospitals voluntarily sent 51 *S. sonnei* isolates to the National Center of Microbiology in Spain, and epidemiologic data were collected from laboratory request forms or by directly contacting the hospitals. From those cases, we identified 37 (72%) suspected cases across 12 autonomous communities (Appendix 1). This finding represented a dramatic increase compared with 2019 data from Spain, in which only 6% of isolates tested in the National Center of Microbiology were identified as MDR (C. Jacqueline et al., unpub. data). We excluded 2020 from review because of the COVID-19 pandemic. 

The median age of the persons with suspected cases was 34 (range 18–75) years (Appendix 2). Seventeen (46%) persons reported diarrhea, and 1 person was asymptomatic (tested as contact of another case); for 19 (51%) persons, symptoms were not reported. Eight (22%) persons were hospitalized for fever, enterocolitis, pancolitis, and dehydration; hospitalization status was unknown for 2 (5%) persons. The percentage of hospitalizations was similar to data from 2018 (20%) but higher than data from 2019 (11%). Mean duration of hospitalization was 5.7 (range 2–10) days, and no person died from their infection. When considered appropriate, patients were treated with ertapenem, meropenem, ciprofloxacin, amoxicillin/clavulanic acid, metronidazole, or fosfomycin (alone or in combination). Antibiotic treatment failure was occasionally observed and resolved by a change in treatment (*7*).

Nineteen (51%) persons from 7 distinct autonomous communities were identified as MSM, and sexual transmission was hypothesized in 4 groups of sexual partners. One person reported sexual contact with persons in France. No person reported exposure to potentially contaminated food or water. Some patients were HIV positive (frequency is omitted to prevent deductive disclosure), and only 2 persons reported using preexposure prophylaxis (18 cases reported not using preexposure prophylaxis; information was unavailable for the 17 other cases).

We performed whole-genome sequencing (WGS; Illumina Inc., https://www.illumina.com) on all isolates from suspected cases. Sequences can be accessed on Enterobase (https://enterobase.warwick.ac.uk; uberstrains ESC_BB1296AA to ESC_BB1332AA). Using the *Escherichia/Shigella* scheme of Enterobase, we performed a core genome multilocus sequence typing analysis. We found 33 (89%) isolates that were within 7 allelic differences (absent alleles were not considered as differences) of the 3 representative outbreak sequences shared by the United Kingdom in the EpiPulse event notification portal (https://www.ecdc.europa.eu/en/publications-data/epipulse-european-surveillance-portal-infectious-diseases). We defined all isolates belonging to that genetic cluster as confirmed cases and belonging to sequence type (ST) 152. Two ST152 isolates showed a high number of allelic differences compared with the main cluster, including 1 isolate from a female case-patient. In addition, we identified 2 isolates as belonging to different sequence types, ST3075 and ST5390 (Appendix 1).

We investigated the genetic diversity of ST152 isolates using a single-nucleotide polymorphism analysis (Center for Genomic Epidemiology, https://cge.food.dtu.dk/services/CSIPhylogeny). We included all the sequences available on Enterobase that corresponded to *S. sonnei* ST152 cases in Spain during 2019–2022. We found a low genetic diversity overall, especially between the sequences of confirmed cases, but we observed 3 phylogenetical clades (confirmed by hierBAPS, https://github.com/gtonkinhill/rhierbaps) (Figure). We observed 1–9 single-nucleotide polymorphisms of difference between cases where sexual transmission was hypothesized.

**Figure:**
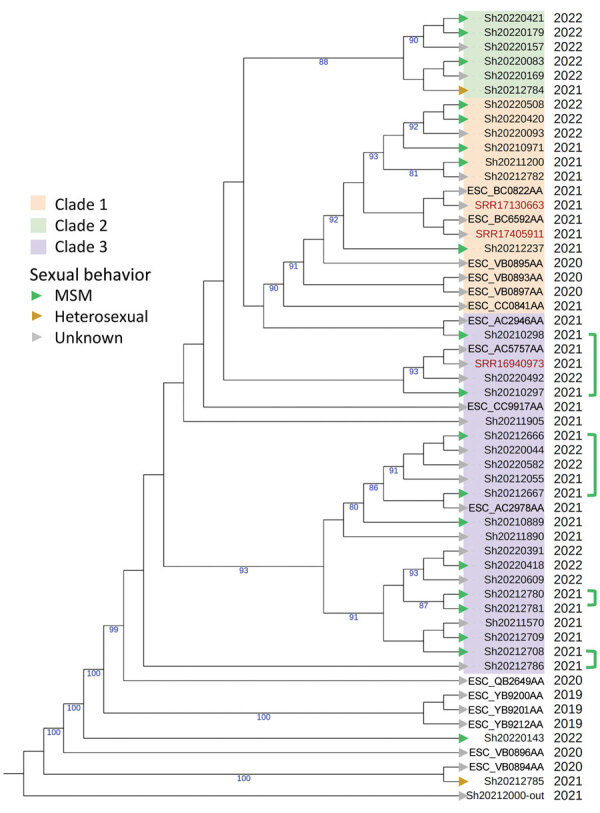
Phylogenetic analysis of ST152 isolates showing 2 clusters from an investigation of suspected multidrug-resistant *Shigella sonnei* in Spain**.** Red text indicates reference sequences from the United Kingdom; blue text indicates assembly barcodes of ST152 feeding sequences available on Enterobase (https://enterobase.warwick.ac.uk) from 2019 to 2022. Green arrowheads indicate cases associated with MSM; green brackets connect known sexual partners. Orange arrowheads indicate cases in heterosexual persons (female or male); gray arrowheads indicate sexual behavior was unknown. A sequence from another sequence type was used as an outgroup. Numbers on branches indicate bootstrap values >80%. MSM, men who have sex with men; ST, sequence type.

We identified the genetic determinants of resistance using PlasmidFinder 2.1 (*8*) and ResFinder 4.1 (*9*,*10*). First, we found that all confirmed cases harbored the plasmid replicon IncFII, which carried the gene *bla*_CTX-M-27_, responsible for resistance to ampicillin, cefepime, cefotaxime, and ceftazidime. However, the ST152 isolate from the female case-patient and the ST3075 and ST5390 isolates harbored different extended-spectrum β-lactamase–producing genes (Appendix Table). Resistance to streptomycin was associated with *aadA5*, resistance to sulfamethoxazole was associated with *sul1*, and resistance to trimethoprim was associated with *dfrA1*, all of which were also harbored by the IncFII plasmid. The gene *mphA* conferring resistance to azithromycin was present in all but 2 isolates. We also detected an additional plasmid replicon, IncB/O/K/Z, in 30 isolates. In the core genome, the same point mutation S83L in the *gyrA* gene, conferring fluoroquinolone resistance, was present in all isolates.

## Conclusion

We describe the circulation in Spain of a cluster of extended spectrum β-lactamase–producing and MDR *S. sonnei* infections genetically related with those observed in a contemporaneous UK outbreak. Because most of the isolates harbored the gene conferring azithromycin resistance, we hypothesize that they would be XDR, even though we did not confirm it phenotypically. We also identified strains of MDR *S. sonnei* that belonged to STs other than the one described in the United Kingdom. This finding raises concerns about the ability to manage the spread of MDR and XDR *Shigella* infections and highlights the need to strengthen surveillance of shigellosis.

In the United Kingdom, sexual transmission between MSM was identified as the main factor of circulation for the strains harboring the IncFII plasmid replicon (*1*). Although we cannot exclude other confounding factors, our results point in the same direction. Indeed, food exposure was not reported, and 19 confirmed case-patients were MSM, even though the respondent rate for sexual orientation was low in our study. However, identifying a female case-patient and a heterosexual male case-patient suggests circulation of MDR *S. sonnei* outside of the MSM population. In Spain, efforts should be made to obtain information related to exposure and to recommend the use of microbiological culture and WGS to identify chains of transmission and antibiotic resistance. Such efforts will be crucial in preventing further selection of antimicrobial resistance, avoiding possible treatment failures, and managing what might become a global outbreak.

Further monitoring of the situation in Spain, as well as in Europe, will be necessary to assess the extent of the circulation of XDR *S. sonnei*. Although more studies are needed to confirm the role of sexual transmission in Spain, communication campaigns, notably in HIV and preexposure prophylaxis clinics, could inform MSM on ways to minimize the risk of infection. Finally, alerting healthcare professionals to the role of sexual transmission in *S. sonnei* infections is critical for obtaining information on sexual history and identifying new cases, particularly in adult men with acute diarrhea.

Appendix 1 Description of cases from study of genetic characterization of extensively drug-resistant *Shigella sonnei* infections, Spain, 2021–2022.

Appendix 2 Additional information for genetic characterization of extensively drug-resistant *Shigella sonnei* infections, Spain, 2021–2022.
