# Supplementary material for: Genetic Characterization of Extensively Drug-Resistant Shigella sonnei Infections, Spain, 2021–2022
Source: Emerg Infect Dis. 2023 Nov;29(11):2370–3. doi: 10.3201/eid2911.221746 (PMC10617328; doi:10.3201/eid2911.221746)
Supplement: Appendix 2 — Additional information for genetic characterization of extensively drug-resistant Shigella sonnei infections, Spain, 2021–2022. [file 22-1746-Techapp-s2.pdf]

Article DOI: https://doi.org/10.3201/eid2911.231746

EID cannot ensure accessibility for supplementary materials supplied by authors. Readers who have difficulty accessing supplementary content should contact the authors for assistance.

Genetic Characterization of Extensively Drug-Resistant *Shigella sonnei* Infections, Spain, 2021–2022

Appendix

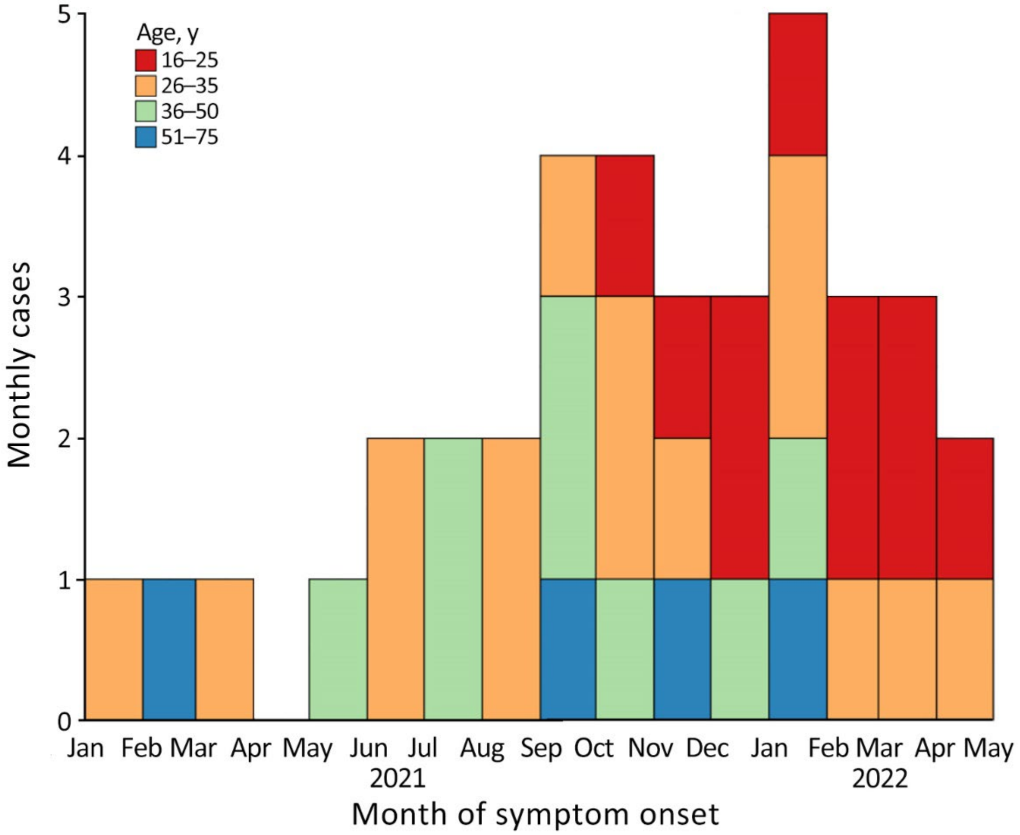

Appendix Figure. Distribution of suspected cases by month-year in Spain (n=37) during January 2021–April 2022 across 4 age categories.
